# A high-throughput quantitative method to evaluate peroxisome-chloroplast interactions in *Arabidopsis thaliana*

**DOI:** 10.3389/fpls.2022.998960

**Published:** 2022-10-20

**Authors:** Kazusato Oikawa, Keiko Midorikawa, Yutaka Kodama, Keiji Numata

**Affiliations:** ^1^ Department of Material Chemistry, Graduate School of Engineering, Kyoto University, Kyoto, Japan; ^2^ Center for Bioscience Research and Education, Utsunomiya University, Utsunomiya, Japan; ^3^ Biomacromolecules Research Team, RIKEN Center for Sustainable Resource Science, Saitama, Japan

**Keywords:** *Arabidopsis thaliana*, chloroplast, confocal laser scanning microscopy (CLSM), distance transformation, peroxisome, organelle interaction

## Abstract

Organelles contribute to plant growth *via* their movements and interactions, which ensure efficient metabolic flow and help plants adapt to environmental stress. Live-cell imaging of the interactions of organelles has been performed in yeast, plant, and animal cells. However, high-throughput quantitative methods are needed to simultaneously analyze the interactions of many organelles in living plant cells. Here, we developed a semi-automatic high-throughput method to quantitatively evaluate the interactions between peroxisomes and chloroplasts using a distance transformation algorithm and high-resolution 3D fluorescent images taken by confocal laser scanning microscopy. Using this method, we measured the 3D distance between the center of peroxisome and chloroplast surface in *Arabidopsis thaliana*. We then compared the distances between these organelles in leaf mesophyll cells under light and dark conditions. This distance was shorter in the light than in the dark, which is in agreement with the findings of previous studies. We used our method to evaluate peroxisome–chloroplast (plastid) interactions in different cell types in the light and dark, including guard, stem, and root cells. Like in mesophyll cells, the distance between the peroxisome and chloroplast was shorter in the light in guard and stem cells, but not in root cells, suggesting that photosynthetic plastids (chloroplasts) play important roles in these interactions. When leaf mesophyll cells were incubated under high-intensity light, the frequency of shorter distances between peroxisomes and chloroplasts significantly increased. Our high-throughput, semi-automatic method represents a powerful tool for evaluating peroxisome–chloroplast interactions in different types of plant cells under various environmental conditions.

## Introduction

Plant organelles dynamically move and frequently interact with other organelles during cytoplasmic streaming (Jaipargas et al., 2016; Barton et al., 2018; Oikawa et al., 2019; Bailli et al., 2020). The movement, positioning, and interactions of organelles are related to plant metabolism, stress conditions, and environmental conditions (Hayashi and Nishimura, 2006; Nyathi and Baker, 2006; Hu et al., 2012; Bailli et al., 2020). For example, peroxisomes actively move using the actin–myosin system (Mano et al., 2002; Mano and Nishimura, 2005; Perico and Sparkes, 2018) and physically interact with chloroplasts (Oikawa et al., 2015; Gao et al., 2016; Oikawa et al., 2019; Bailli et al., 2020). In the light, peroxisomes change from spherical to elliptical (elongated) and strengthen their interactions with chloroplasts (Oikawa et al., 2015). Because peroxisomes and chloroplasts share several metabolic pathways, such as photorespiration and the jasmonic acid pathway (Hayashi and Nishimura, 2006; Nyathi and Baker, 2006; Hu et al., 2012), their interactions appear to regulate metabolic pathways. The peroxisome–chloroplast interaction has been studied using electron microscopy and confocal laser scanning microscopy (CLSM) (Frederick and Newcomb, 1969; Tolbert and Essner, 1981; Oikawa et al., 2015; Oikawa et al., 2019; Bailli et al., 2020). The interaction strength between peroxisomes and chloroplasts has been measured and quantified using a femtosecond laser (Oikawa et al., 2015) and optical tweezers (Gao et al., 2016). However, it remains difficult to perform high-throughput evaluation of multiple peroxisome–chloroplast interactions in a plant cell.

A high-throughput method was previously developed to evaluate organelle interactions with ultra-resolution microscopy and electron microscopy. This method automatically calculates the distance between two organelles labeled with fluorescent proteins *via* a process known as distance transformation (Belevich et al., 2016). The distance transformation method has been used to measure the distances between endosomes and mitochondria in living animal cells *via* ultra-resolution microscopy (Das et al., 2016) and the distance between a sperm cell and vacuole and a lipid body in a fixed pollen tube *via* electron microscopy (Akita et al., 2021). In the present study, we developed a high-throughput method based on distance transformation and CLSM to evaluate peroxisome–chloroplast interactions *via* semi-automatic measurement of the minimum distance between the chloroplast surface and the center of peroxisome. Using this method, we successfully evaluated numerous peroxisome–chloroplast interactions in *Arabidopsis thaliana* cells simultaneously.

## Material and methods

### Plant materials and growth conditions

*Arabidopsis* (*Arabidopsis thaliana*, At) Columbia accession (Col-0) expressing GFP-PTS1 was used to visualize peroxisomes (Mano et al., 2002). The plants were grown on 0.8% agar plates containing half-strength Murashige and Skoog medium with 1% sucrose (pH 5.7) in an incubator (Nihonika, Japan) under a 16-h-light/8-h-dark cycle at 23°C after vernalization. The light intensity was 100 µmol m^−2^ s^−1^. We used 2- to 3-week-old plants in each experiment.

### Imaging analysis

To capture fine three-dimensional images (X-, Y-, and Z-axes), we used a confocal laser scanning microscope (CLSM, Zeiss LSM880) with a 63× oil immersion objective (Plan-Apochromat 63×/1.4 Oil DIC M27) and a zoom lens (×2.5) in Airy scan mode (Carl Zeiss, Jena, Germany). An argon laser was used for excitation of GFP and chlorophyll with the following excitation/emission wavelengths: 488 nm/507–525 nm for GFP fluorescence and 480 nm/660–700 nm for chlorophyll autofluorescence. Z-axis sections were taken every 0.5 µm, with a total thickness of at least 15 µm, to include chloroplasts and peroxisomes at periclinal cell walls. The three-dimensional images were stacked into a 3D file using Imaris (v8.4.1, Bitplane). All images were obtained as 488 × 488 pixel size, and saved as tiff files.

Light-adapted adult rosette leaves, stems, and roots were prepared for observation after deaeration and placed on a glass coverslip with pure water. To prepare samples under light conditions, we incubated the tissues under 10 µmol m^−2^ s^−1^ light for 1 h. To prepare samples under dark conditions, light-adapted samples were transferred to the dark for 30 min. For the 3D reconstruction of the single channel of GFP-PTS1 (peroxisome) and chlorophyll autofluorescence (Chloroplast) was reconstructed as the Surface/Spots objects using the Surface/Spots module in Imaris, respectively. To measure the distance of nearest spot object (peroxisome) from the surface object (chloroplast), XTension distance transform module in Imaris was used. The nearest distance from the center of each spot to the border of each surface object was measured by creation of new channel which showed the intensity exhibiting the distance information. Each value was exported and analyzed by comparison of all values of each group.

### Light irradiation and H_2_O_2_ treatment

To treat leaf cells with light, a rosette leaf sample mounted on a coverslip was irradiated with 200 and 1,000 µmol m^−2^ s^−1^ light for 2 h using light-emitting diodes (CCS, Japan). After the irradiation, we captured z-stack images of peroxisomes and chloroplasts using CLSM. To treat leaf cells with hydrogen peroxide (H_2_O_2_), a rosette leaf sample was deaerated with pure water, submerged in 3% H_2_O_2_ solution in a 1.5-mL tube, and incubated for 30 min under 100 µmol m^−2^ s^−1^ white light. Following incubation, the leaf sample was placed on a glass coverslip with pure water for CLSM analysis.

### Statistical analysis

Distances between peroxisomes and chloroplasts were automatically calculated with Imaris software. Values >3.5 µm were removed based on the radius of a chloroplast (**Supplementary Figure 1**) to avoid incorrect measurements across different chloroplasts. After the data set was opened in Excel (Microsoft), we counted the number of peroxisome–chloroplast interactions with distances of 0–3.5, 0–0.5, and 0.5–1.0 µm. The frequency of peroxisome–chloroplast interactions at short distances (0–0.5 and 0.5–1.0 µm) was calculated by dividing the number of interactions at a distance of 0–0.5 or 0.5–1.0 µm by the total number of interactions at a distance of 0–1.0 µm. At least three different experiments were performed for statistical analysis. Box plot graphs were generated using Igor64 (WaveMetrics) with Excel datasheets (Microsoft).

## Results

### Basic procedure to evaluate peroxisome-chloroplast interactions using the distance transformation method

To evaluate a large number of peroxisome–chloroplast interactions in a CLSM image, we measured the shortest distance between peroxisomes and chloroplasts using a distance transformation algorithm within Imaris software (Bitplane). Using CLSM in airy scan mode (Zeiss, Germany), we took fine three-dimensional images of peroxisomes and chloroplasts in cells. After integrating the images into Imaris software, we adjusted the parameters of the software to measure the sizes of peroxisomes and chloroplasts and to define the center of peroxisome and chloroplast surface (**Supplementary Figure 2**). By using the distance transformation tool within Imaris, the shortest distance between the center of peroxisome and chloroplast surface was automatically measured. To determine the accuracy of the automatic measurements using distance transformation, we manually checked all the distances (**Supplementary Figure 3**). During manual checking, we removed the redundant distance data selected due to abnormal peroxisome morphology, such as peroxules (Sinclair et al., 2009). Based on the radius of a peroxisome (**Supplementary Figure 4**), we classified the resulting distance data into two categories: 0–0.5 µm (positive interaction) and 0.5–1.0 µm (negative interaction). Using this procedure, we detected approximately 800 interactions between peroxisomes and chloroplasts in a single CLSM image.

### Evaluation of light-dependent peroxisome–chloroplast interactions using the distance transformation method

**Figure 1:**
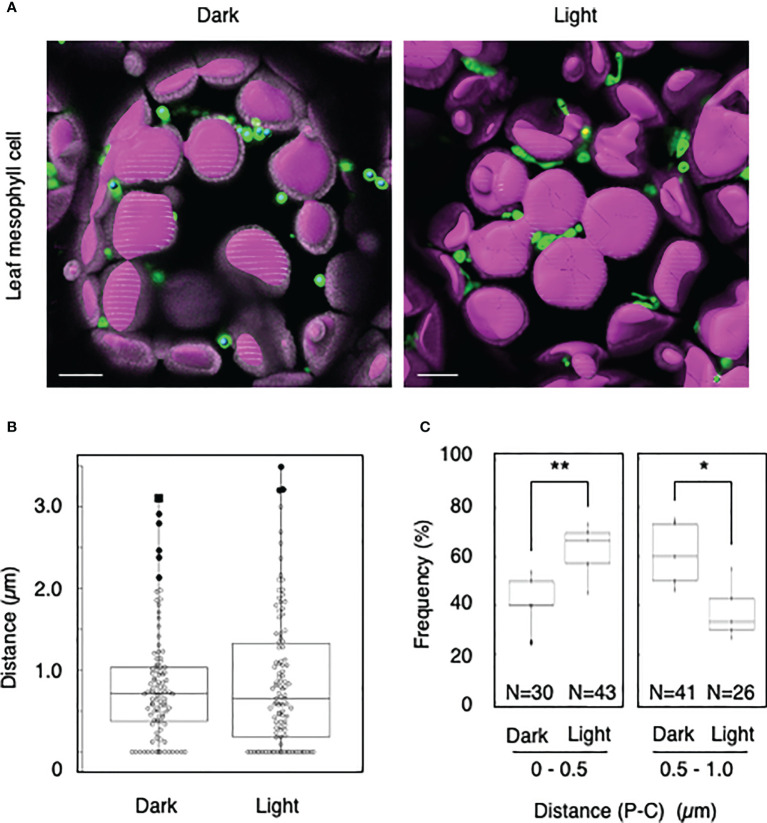
Measurement of the distances between chloroplasts and peroxisomes in leaf mesophyll cells. **(A)** Representative images of chloroplasts (magenta) and peroxisomes (green) in leaf mesophyll cells in the dark and light. Bars, 10 µm. **(B)** Box plots of the distances between peroxisomes and chloroplasts in the dark and light from five cells. The average distance was 0.81 ± 0.68 µm (n = 99) in the dark and 0.51 ± 0.46 (n = 106) in the light. The median was 0.72 µm in the dark and 0.657 µm in the light. **(C)** Frequency of the distances between peroxisomes and chloroplasts in the dark and light. The frequency of distances of 0–0.5 µm was 41.8 ± 11.2% (n = 30) in the dark and 62.4 ± 11.5% (n = 43) in the light. The frequency of distances of 0.5–1.0 µm was 60.9 ± 13.1% (n = 41) in the dark and 37.6 ± 11.5% (n = 26) in the light in **(B)**. *P < 0.01, **P < 0.01, Student’s *t*-test.

We tested whether the distance transformation method could be used to evaluate peroxisome–chloroplast interactions in *Arabidopsis* cells. Because light-dependent peroxisome–chloroplast interactions in mesophyll cells have been described (Oikawa et al., 2015), we initially applied our method to mesophyll cells in the dark and light (**Figures 1A, B**). In the dark, distances of 0–0.5 µm were less frequent than distances of 0.5–1.0 µm (**Figure 1C**). By contrast, in the light, distances of 0–0.5 µm were more frequent than distances of 0.5–1.0 µm (**Figure 1C**). The results of examining light-dependent peroxisome–chloroplast interactions are consistent with previous findings (Oikawa et al., 2015). We therefore conclude that the distance transformation method is suitable for evaluating peroxisome–chloroplast interactions in *Arabidopsis* cells.

### Peroxisome–chloroplast interactions in cells containing mature chloroplasts

**Figure 2:**
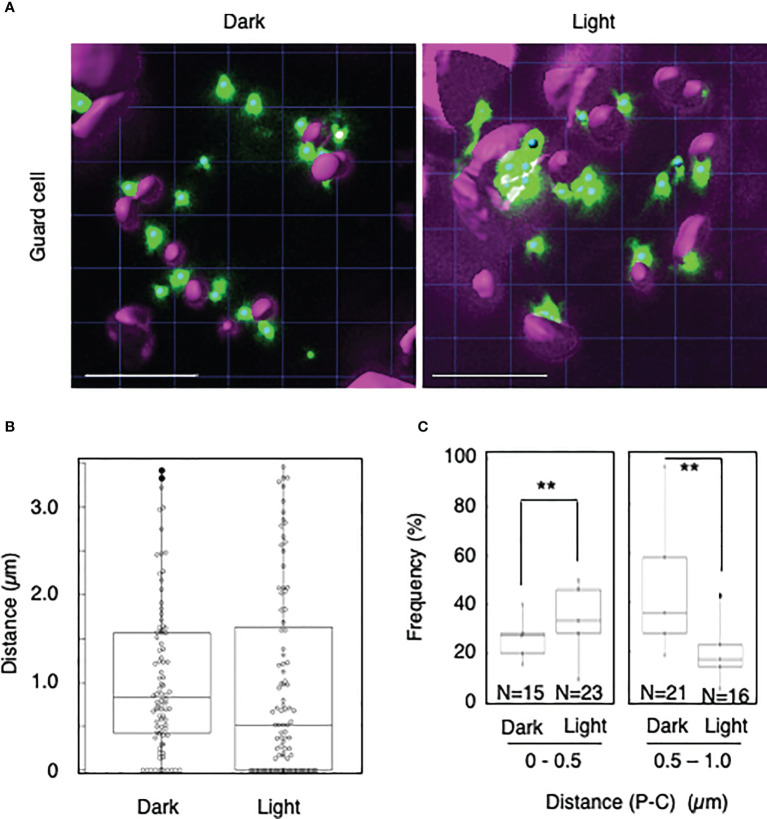
Measurement of the distances between chloroplasts and peroxisomes in guard cells. **(A)** Representative images of chloroplasts (magenta) and peroxisomes (green) in guard cells in the dark (left) and light (right). Bars, 10 µm. **(B)** Box plots of the distances between peroxisomes and chloroplasts in guard cells in the dark and light from five cells. The average distance was 1.05 ± 0.87 µm (n = 88) in the dark and 1.00 ± 1.06 (n = 92) in the light. The median distance was 0.83 µm in the dark and 0.61 µm in the light. **(C)** Frequency of the distances between peroxisomes and chloroplasts in the dark and light. The frequency of distances of 0–0.5 µm was 37.7 ± 16.0% in the dark (n = 15) and 62.3 ± 16.0% (n = 23) in the light, whereas the frequency of distances of 0.5–1.0 µm was 59.5 ± 11.6% (n = 21) in the dark and 40.5 ± 11.6% (n = 16) in the light in **(B)**. **P < 0.01, Student’s *t*-test.

**Figure 3:**
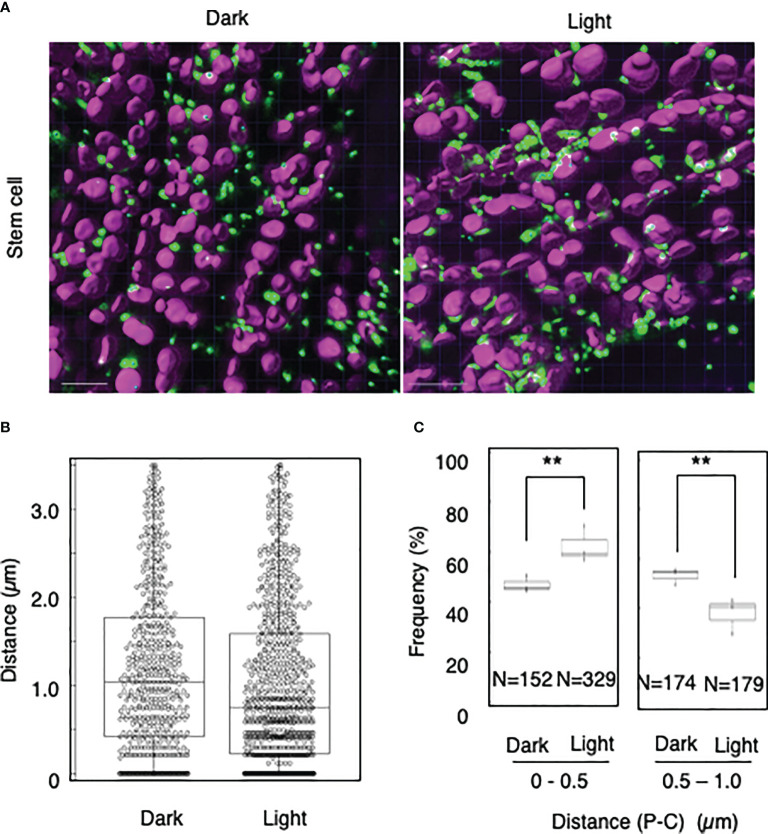
Measurement of the distances between chloroplasts and peroxisomes in stem cells. **(A)** Representative images of chloroplasts (magenta) and peroxisomes (green) in stem cells in the dark (left) and light (right). Bars, 10 µm. **(B)** Box plots of the distances between peroxisomes and chloroplasts in stem cells in the dark and light from three image areas. The average distance was 1.20 ± 0.94 µm (n = 540) in the dark and 1.01 ± 0.92 (n = 844) in the light. The median distance was 1.04 µm in the dark and 0.75 µm in the light. **(C)** The frequency of the distances between peroxisomes and chloroplasts in the dark and light. The frequency of distances of 0–0.5 µm was 46.9 ± 3.3% in the dark (n = 152) and 62.3 ± 7.2% in the light (n = 329), whereas the frequency of distances of 0.5–1.0 µm was 53.5 ± 3.3% in the dark (n = 174) and 37.7 ± 7.2% in the light (n = 179) in **(B)**. **P < 0.01, Student’s *t*-test.

**Figure 4:**
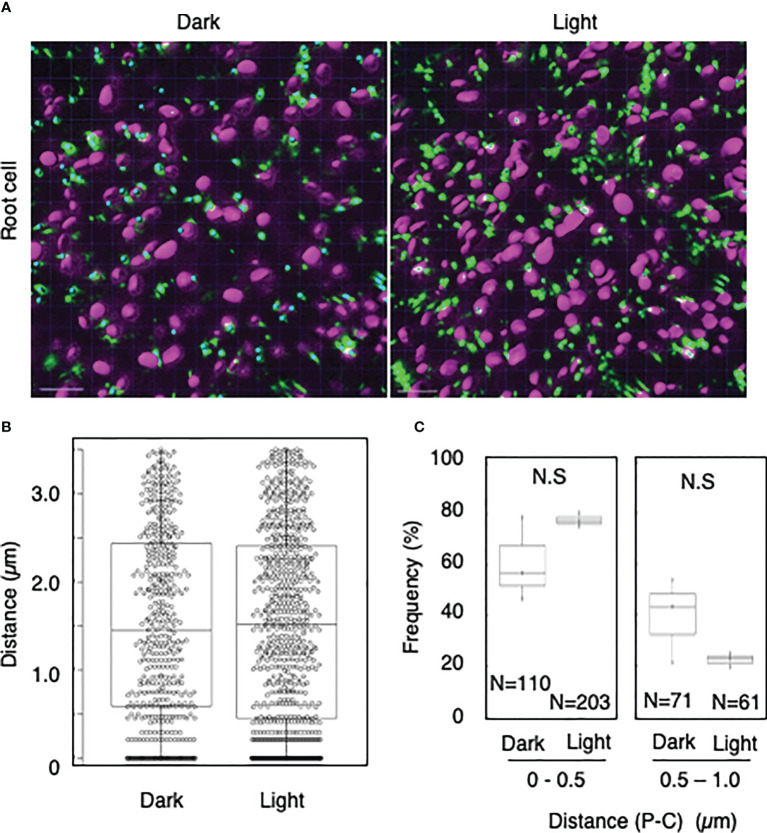
Measurement of the distances between chloroplasts and peroxisomes in root cells. **(A)** Representative images of chloroplasts (magenta) and peroxisomes (green) in greening root cells in the dark (left) and light (right). Bars, 10 µm. **(B)** Box plots of the distances between peroxisomes and chloroplasts in greening root cells in the dark and light from three image areas. The average distance was 1.52 ± 1.07 µm (n = 483) in the dark and 1.52 ± 1.09 (n = 761) in the light. The median distance was 1.45 µm in the dark and 1.52 µm in the light. **(C)** Frequency of the distances between peroxisomes and chloroplasts in the dark and light. The frequency of distances of 0.5–1.0 µm was 60.7 ± 16.6% (n = 110) in the dark and 77.7 ± 2.9% (n = 203) in the light, whereas the frequency of distances of 0.5–1.0 µm was 39.3 ± 16.8% (n = 71) in the dark and 37.7 ± 7.2% (n = 61) in the light in **(B)**. N.S indicates no significant difference between the dark and light.

To explore whether light-dependent peroxisome–chloroplast interactions occur in various types of *Arabidopsis* cells, we applied the distance transformation method to guard, stem, and root cells in the dark and light (**Figures 2**–**4**). Like in mesophyll cells, mature chloroplasts are well developed in guard and stem cells, but not in root cells. After adapting *Arabidopsis* plants to the dark or light, we randomly selected more than five cells from guard cells, stems, or root tissues (**Figures 2**–**4**). In guard and stem cells, we observed a similar trend to that in leafmesophyll cells; more frequent (guard cells: 38% in the dark and 62% in the light, and stem cells: 47% in the dark and 62% in the light), whereas interactions at a distance of 0.5–1.0 μm were less frequent (guard cells: 60% in the dark and 41% in the light, and stem cells: 54% in the dark and 38% in the light) (**Figures 2**, **3**). We then examined peroxisome–chloroplast (plastid) interactions in root cells using the distance transformation method (**Figure 4**). To determine the positions of plastids (immature chloroplasts), we selected root cells around the upper region of the main root of plants grown on agar plates in white light because the plastids in these cells exhibited slight chlorophyll autofluorescence (**Figure 4A**). However, there was no clear difference in the frequency of the distances between plants grown in the dark and light (0–0.5 µm: 57% in the dark and 78% in the light, 0.5–1.0 µm: 39% in the dark and 22% in the light) (**Figures 4B, C**). These results indicate that the distance transformation method with CLSM can be used to evaluate peroxisome–chloroplast interactions in various types of *Arabidopsis* cells. Moreover, peroxisome–chloroplast interactions appear to be induced in mature chloroplasts irradiated by light.

### The peroxisome–chloroplast interaction is dependent on light intensity

**Figure 5:**
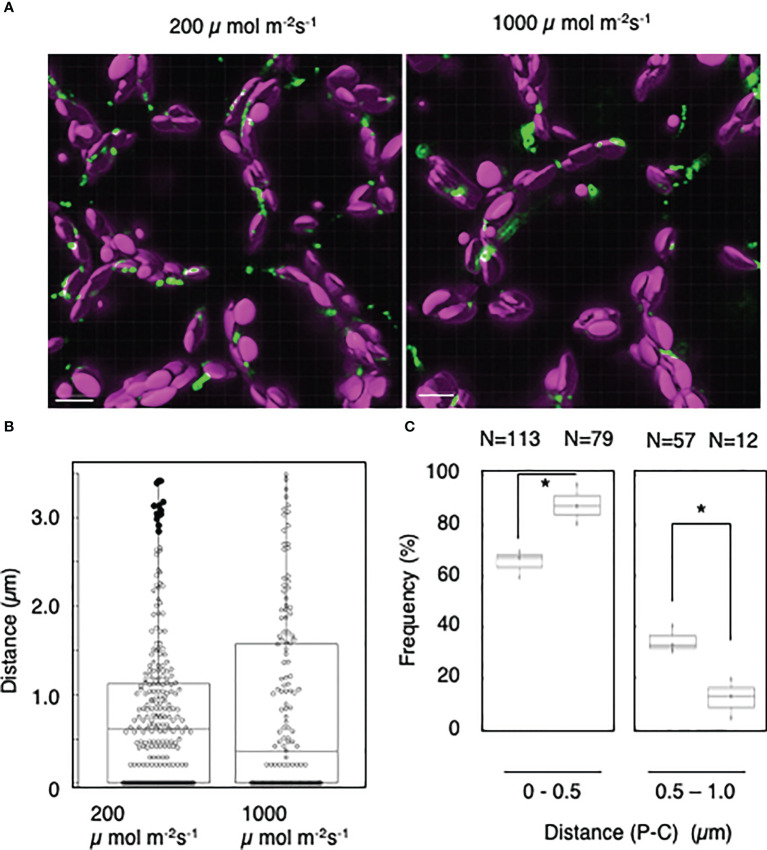
Measurement of the distances between chloroplasts and peroxisomes in leaf mesophyll cells under high-light conditions. **(A)** Representative images of chloroplasts (magenta) and peroxisomes (green) in leaf mesophyll cells adapted to low-intensity (200 µmol m^−2^ s^−1^, left) and high-intensity (1,000 µmol m^−2^ s^−1^, right) light for 2 h. Bars, 10 µm. **(B)** Box plots of the distances between peroxisomes and chloroplasts in **(A)**. The average distance was 0.79 ± 0.84 µm (n = 252) in 200 µmol m^−2^ s^−1^ light and 0.829 ± 1.01 µm (n = 143) in 1,000 µmol m^−2^ s^−1^ light from five cells. The median was 0.62 µm in 200 µmol m^−2^ s^−1^ light and 0.37 µm in 1,000 µmol m^−2^ s^−1^ light. Frequency of the distances between peroxisomes and chloroplasts in **(A)**. The frequency of distances of 0–1.0 µm between peroxisomes and chloroplasts. Bars, 10 µm. **(C)** The frequency of distances of 0–0.5 µm between peroxisomes and chloroplasts in 200 µmol m^−2^ s^−1^ and 1,000 µmol m^−2^ s^−1^ light was 65.6 ± 5.38% (n = 113) in the dark and 88.1 ± 7.85% (n = 79) in the light, whereas the frequency of distances of 0.5–1.0 µm was 34.5 ± 5.38% (n = 57) in the dark and 12.0 ± 7.85% (n = 12) in the light in **(B)**. *P < 0.01, Student’s *t*-test.

To further analyze the light-induced interactions of mature chloroplasts with peroxisomes, we applied the distance transformation method to mesophyll cells incubated under low- or high-intensity light (200 or 1,000 µmol m^−2^ s^−1^, respectively) (**Figure 5A**). When we measured the distance between peroxisomes and chloroplasts (**Figure 5B**), the frequency of interactions at a distance of 0–0.5 µm was dramatically higher in cells incubated under high-intensity light: 66% under 200 µmol m^−2^ s^−1^ of light and 88% under 1,000 µmol m^−2^ s^−1^ of light (**Figure 5C**). By contrast, the frequency of interactions at a distance of 0.5–1.0 was lower under high-intensity light: 35% under 200 µmol m^−2^ s^−1^ light and 12% under 1,000 µmol m^−2^ s^−1^ light (**Figure 5C**). These results suggest that mature chloroplasts interact with peroxisomes in a light intensity–dependent manner.

## Discussion

In this study, we developed a semi-automatic method to evaluate a large number of peroxisome–chloroplast interactions in living plant cells by CLSM using the distance transformation algorithm. Using the method, we succeeded in evaluating peroxisome–chloroplast interactions in various types of *Arabidopsis* cells such as leaf mesophyll, guard, stem, and root cells. We also found that peroxisome–chloroplast interactions in mesophyll cells are induced in a light intensity–dependent manner.

To date, peroxisome–chloroplast interactions have been evaluated using several tools, such as electron microscopy, CLSM, femtosecond lasers, and optical tweezers (Sinclair et al., 2009; Oikawa et al., 2015; Gao et al., 2016). These techniques successfully identified peroxisome–chloroplast interactions in plant cells. However, using these techniques, peroxisome–chloroplast interactions are manually evaluated one-by-one, resulting in low-throughput analysis. To increase the throughput, in the present study, we employed the distance transformation algorithm to evaluate a large number of peroxisome–chloroplast interactions in living cells. With this semi-automatic algorithm, we automatically measured the shortest distance between the chloroplast surface and the center of peroxisome and evaluated peroxisome–chloroplast interactions by combining this information with that about the radiuses of chloroplasts and peroxisomes (**Supplementary Figures 1** and **4**). We simultaneously evaluated over 800 interactions between chloroplasts and peroxisomes, achieving high-throughput analysis of these interactions in living plant cells (**Figures 1**–**5**). Therefore, the distance transformation method is a good technique for exploring peroxisome–chloroplast interactions.

Our study revealed light-dependent differences in peroxisome–chloroplast interactions in mesophyll, guard, and stem cells (**Figures 1**–**3**), as previously observed in mesophyll cells (Oikawa et al., 2015). By contrast, light-dependent differences in peroxisome–chloroplast interactions were not observed in root cells (**Figure 4**). Because root cells do not contain mature chloroplasts, we expect that light-dependent peroxisome–chloroplast interactions occur only in cells containing mature chloroplasts. Indeed, a previous study revealed that light-dependent peroxisome–chloroplast interactions decreased in response to treatment with photosynthetic electron transport inhibitors and in mutant cells lacking photosynthetic activity (Oikawa et al., 2015). Yet, peroxisomes in root cells were shown to have different characteristics from peroxisomes in seed and leaf cells (Nyati and Baker, 2006). Given this observation, the characteristics of peroxisomes might also be involved in light-dependent peroxisome–chloroplast interactions. Further study is needed to understand light-dependent peroxisome–chloroplast interactions.

In this work, we also determined that peroxisome–chloroplast interactions increased in response to high-intensity light (1,000 µmol m^−2^ s^−1^) (**Figure 5**). High-intensity light conditions should promote photosynthesis and photorespiration in cells, perhaps leading to increased peroxisome–chloroplast interactions. High-intensity light increases reactive oxygen species production in photosynthetic cells (Halliwell and Gutteridge, 1984), and peroxisomes showed aberrant morphology and interacted with chloroplasts in *Arabidopsis* cells treated with H_2_O_2_ (Sinclair et al., 2009). Indeed, using the distance transformation method, we confirmed that H_2_O_2_ induces peroxisome–chloroplast interactions in *Arabidopsis* mesophyll cells (**Supplementary Figure 5**). Taken together, increased reactive oxygen species production *via* excess photosynthetic reactions under high-intensity light might promote peroxisome–chloroplast interactions.

Using the distance transformation method, we evaluated peroxisome–chloroplast interactions in *Arabidopsis* cells. This method could possibly be used to evaluate the interactions of other organelles in various types of living cells, including plant and animal cells, in different environments. To precisely evaluate the physical interactions between two organelles in living plant cells, the distances between organelles whose membranes are marked by specific probes or fluorescent proteins could be measured using advanced methods.

Our findings indicate that the distance transformation method is a powerful tool for evaluating the interactions between two different types of organelles in living cells.

## Data availability statement

The original contributions presented in the study are included in the article/**Supplementary Material**. Further inquiries can be directed to the corresponding authors.

## Author contributions

KO, YK, and KN designed the study. KO and KM performed experiments. KO took CLSM images. All the authors analyzed the data and wrote the manuscript. All authors contributed to the article and approved the submitted version.

## Funding

This work was supported by the Japan Science and Technology Agency Exploratory Research for Advanced Technology program (JST-ERATO; Grant Number JPMJER1602).

## Acknowledgments

We thank Dr. Shoji Mano (National Institute for Basic Biology, Japan) for providing transgenic *Arabidopsis* expressing GFP-PTS1.

## Conflict of interest

The authors declare that the research was conducted in the absence of any commercial or financial relationships that could be construed as a potential conflict of interest.

## Publisher’s note

All claims expressed in this article are solely those of the authors and do not necessarily represent those of their affiliated organizations, or those of the publisher, the editors and the reviewers. Any product that may be evaluated in this article, or claim that may be made by its manufacturer, is not guaranteed or endorsed by the publisher.
