# Supplementary material for: A high-throughput quantitative method to evaluate peroxisome-chloroplast interactions in Arabidopsis thaliana
Source: Front Plant Sci. 2022 Oct 20;13:998960. doi: 10.3389/fpls.2022.998960 (PMC9632854; doi:10.3389/fpls.2022.998960)
Supplement: Supplementary file 1 [file DataSheet_1.pdf]

Supplementary Figures

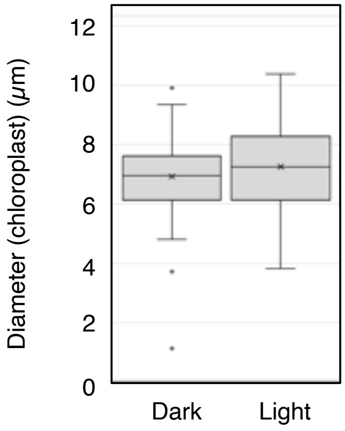

Average diameter of chloroplast in dark and light

	Dark	Light
Length (μm)<br>(SD)	6.96<br>(1.12)	7.27<br>(1.47)

Supplementary Figure 1. Radius of the chloroplast. More than 80 images of chloroplasts were captured using confocal laser scanning microscopy (CSLM) from five different cells after adaptation to dark or light conditions. The long- and short-axis lengths of chloroplasts were measured using ImageJ to calculate the radius of the chloroplast: $\text{Radius} = (\text{Long axis length} + \text{short axis length})/2$.

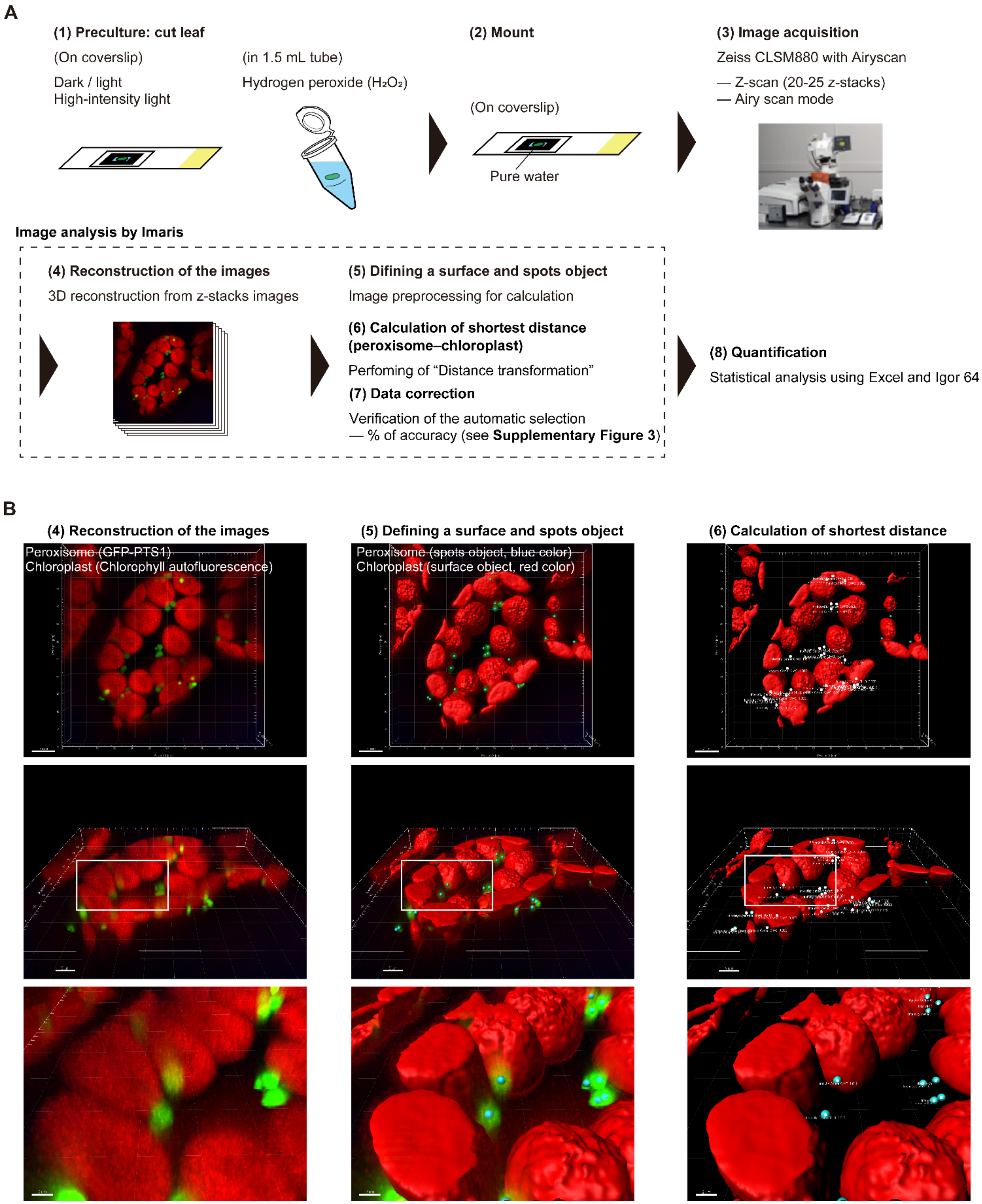

Supplementary Figure 2. Procedure for measuring interactions between peroxisomes and chloroplasts (distance transformation). (**A**) Overview of the distance transformation method from sample preparation to data analysis. (1) Preculture of a sample: A cut rosette leaf section is placed on a glass coverslip in pure water after deaeration. The leaf section is incubated in 10 μmol m^{-2} s^{-1} light

for 1 h (light treatment) and transferred to the dark for 30 min (dark treatment). For hydrogen peroxide (H_2O_2) treatment, the cut leaf is submerged in a 1.5-mL tube filled with 3% H_2O_2 for 30 min in the light and mounted on a glass coverslip (2). (3) Acquisition setting of LSM880 with Airy Scan (Zeiss, Germany) as a z-series for 3D construction for capturing fine images. (4-6) To obtain the shortest distance between peroxisomes and chloroplasts, each parameter is set for the distance transformation methods in Imaris software (bitplane; https://imaris.oxinst.com/), which extracts the chloroplast surface and center of the peroxisome. (6) All distances between 0 and 3.5 μm are collected. (7) Verifying whether the short distance is correctly defined by comparing the results with original images. (8) The values are classified into two types, 0–0.5 μm and 0.5–1.0 μm, and the frequency of each type is calculated. Box plots are constructed using Igor64 (WaveMetrics) based on information in Excel datasheets. (**B**) Analytical images in procedures (4) to (6) shown in (**A**). The upper row is an orthogonal view, the middle row is an oblique view, and the lower row is an enlarged view of the white rectangular area shown in the middle row. Surface objects (red, chloroplasts) and spots objects (blue, peroxisomes) were determined based on 3D images reconstructed from z-stacks of fluorescence images. A "distance transformation" was performed to obtain the distance data at each spot. Bars are 5 μm (the upper and middle rows) and 2 μm (the lower row), respectively.

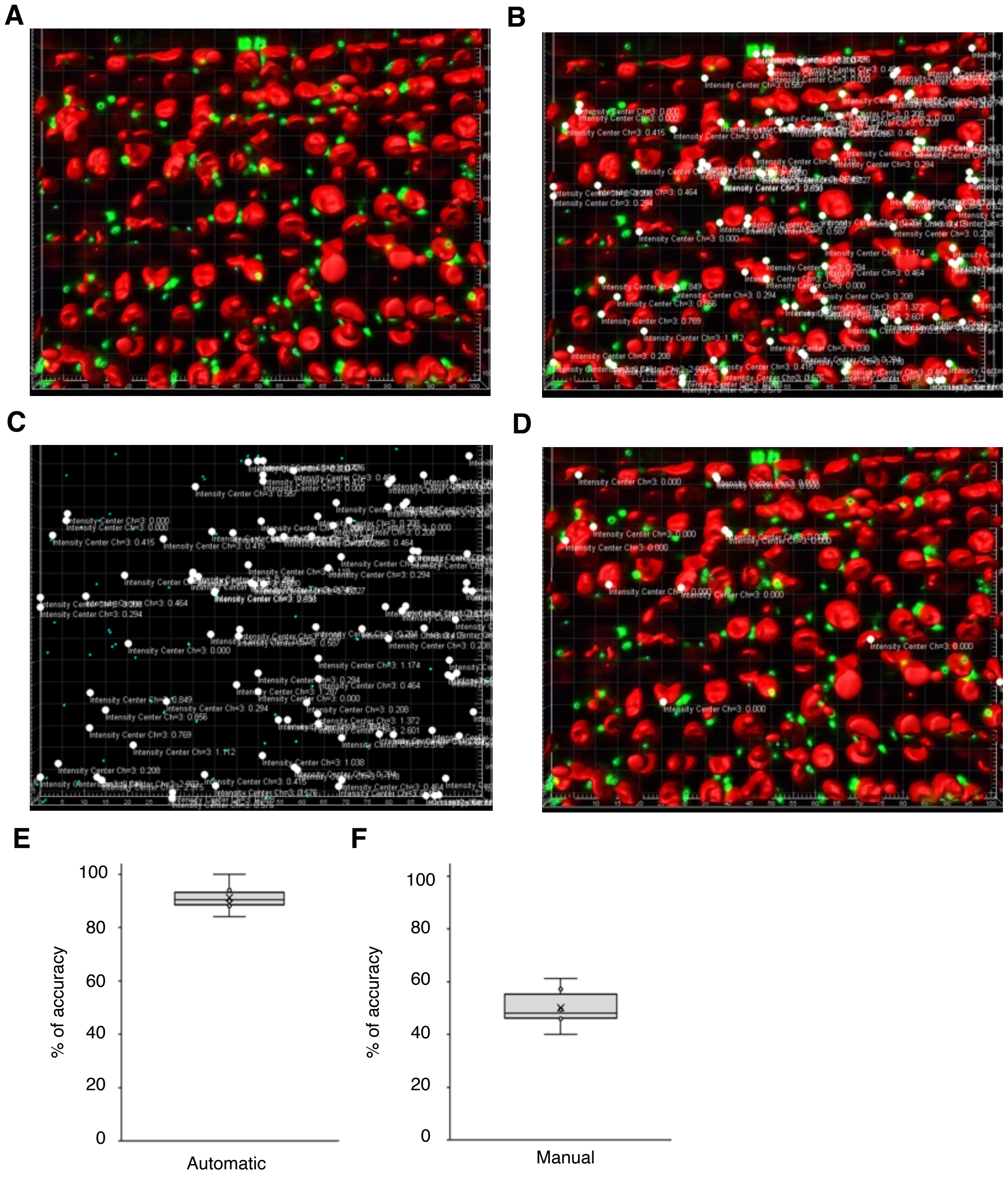

Supplementary Figure 3. Verification of the distance transformation method. (**A-D**) Images of the surfaces of chloroplasts (red) and centers of peroxisomes (green) marked by the distance transformation tool in Imaris software. (**B**) Representative images of the chloroplasts and peroxisomes marked with white points, representing a 0-µm distance between peroxisomes and chloroplasts. (**C**) The centers of peroxisomes and the points in (**B**). (**D**) Incorrect points are represented. (**E and F**) The % accuracy of the automatic (**E**) and manual (**F**) methods.

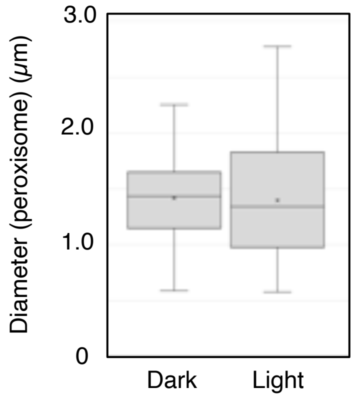

Average diameter of peroxisome in dark and light

	Dark	Light
Length (μm)<br>(SD)	1.42<br>(0.39)	1.40<br>(0.50)

Supplementary Figure 4. Radius of the peroxisome. More than 80 images of peroxisomes were captured using confocal laser scanning microscopy (CSLM) from five different cells after adaptation to dark or light conditions. A The long- and short- axis lengths of peroxisomes were measured using Image J for to calculation of the radius of the chloroplast:; Radius=(Long axis length + short axis length)/2.

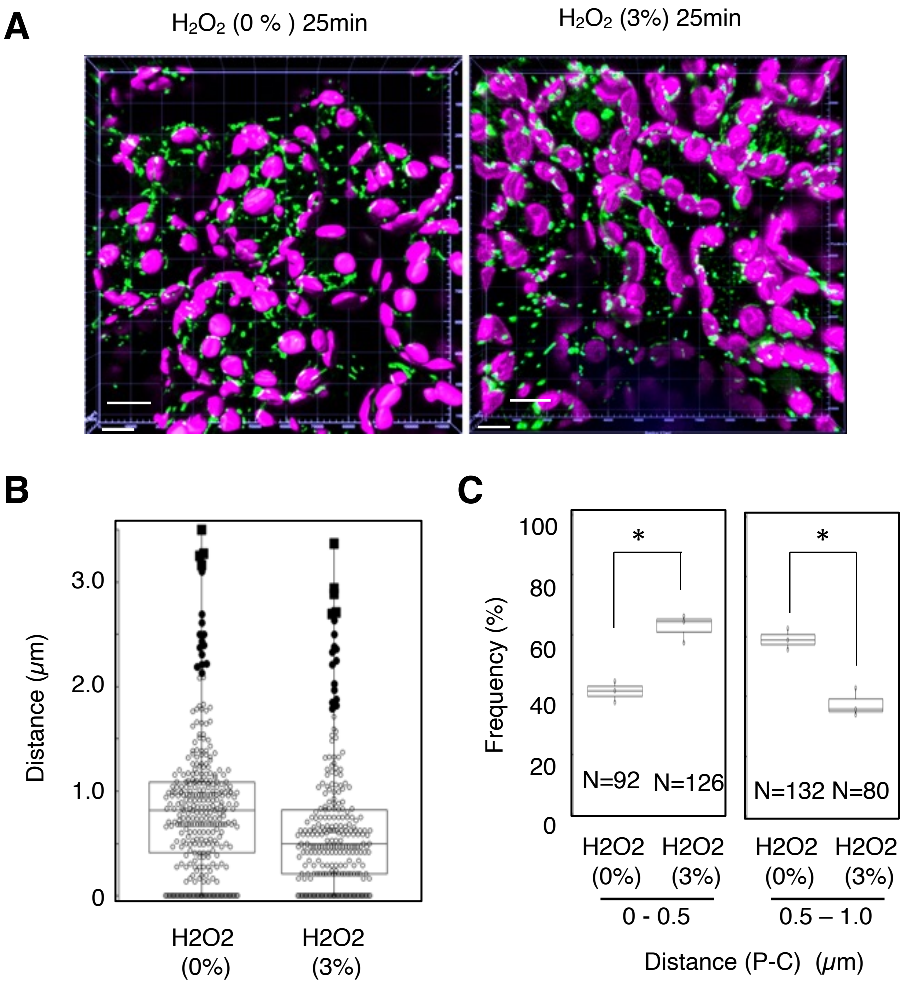

Supplementary Figure 5. Measurement of the distances between chloroplasts and peroxisomes in leaf mesophyll cells under H_2O_2 treatment. (**A**) Representative images of chloroplasts (magenta) and peroxisomes (green) in leaf mesophyll cells treated with 0% (left, control) and 3% (right) H_2O_2 for 25 min. Bars, 10 μm. (**B**) Box plots of the distances between peroxisomes and chloroplasts in (A). The average distance was 0.84 ± 0.64 μm (n = 325) under control conditions and 0.64 ± 0.62 (n = 254) following 3% H_2O_2 treatment with five cells. The median was 0.82 μm under control conditions and 0.50 μm under 3% H_2O_2 treatment. (**C**) Frequency of the distances between peroxisomes and chloroplasts under control conditions and 3% H_2O_2 treatment. The frequency of distances of 0–0.5 μm was 41.0 ± 3.60% (n = 92) in the dark and 62.5 ± 4.70% (n = 126) in the light, whereas the frequency of distances of 0.5–1.0 μm was 59.0 ± 3.6% (n = 132) in the dark and 37.5 ± 4.70% (n = 80) in the light in (**B**). *$P < 0.01$, Student's *t*-test.
